# Liver Immune Cells Release Type 1 Interferon Due to DNA Sensing and Amplify Liver Injury from Acetaminophen Overdose

**DOI:** 10.3390/cells7080088

**Published:** 2018-07-27

**Authors:** Alan Moreira de Araujo, Maísa Mota Antunes, Matheus Silvério Mattos, Ariane Barros Diniz, Débora Moreira Alvarenga, Brenda Naemi Nakagaki, Érika de Carvalho, Viviane Aparecida Souza Lacerda, Raquel Carvalho-Gontijo, Jorge Goulart, Kassiana Mafra, Maria Alice Freitas-Lopes, Hortência Maciel de Castro Oliveira, Camila Miranda Dutra, Bruna Araújo David, Aristóbolo Mendes Silva, Valerie Quesniaux, Bernhard Ryffel, Sergio Costa Oliveira, Glen N. Barber, Daniel Santos Mansur, Thiago Mattar Cunha, Rafael Machado Rezende, André Gustavo Oliveira, Gustavo Batista Menezes

**Affiliations:** 1 Center for Gastrointestinal Biology, Departamento de Morfologia, Instituto de Ciências Biológicas, Universidade Federal de Minas Gerais, Av Antonio Carlos, 6627-Belo Horizonte, Minas Gerais 31270-901, Brazil; amoreiradearaujo@gmail.com (A.M.d.A.); maisaantunes@gmail.com (M.M.A.), mattosms@yahoo.com.br (M.S.M.); abarrosdiniz@gmail.com (A.B.D.); deboraalvarenga@yahoo.com (D.M.A.); brendanaemi22@gmail.com (B.N.N.); erikacarvalhobio@gmail.com (É.d.C.); viviane_lacerda@outlook.com (V.A.S.L.); rakel.cg@gmail.com (R.C.-G.); jorge.ferreira@cpqrr.fiocruz.br (J.G.); kassiana93@gmail.com (K.M.); licefl95@gmail.com (M.A.F.-L.); hortsmaciel@gmail.com (H.M.d.C.O.); camiladutra_mm@hotmail.com (C.M.D.); brunaraujodavid@gmail.com (B.A.D.); 2 Departamento de Morfologia, Instituto de Ciências Biológicas, Universidade Federal de Minas Gerais, Belo Horizonte, Minas Gerais 31270-901, Brazil; aristobolosilva@gmail.com; 3 Experimental and Molecular Immunology and Neurogenetics CNRS, University of Orleans, 45000 Orleans, France; quesniaux@cnrs-orleans.fr (V.Q.); bernhard.ryffel@cnrs-orleans.fr (B.R.); 4 Departamento de Bioquímica e Imunologia, Instituto de Ciências Biológicas, Universidade Federal de Minas Gerais, Belo Horizonte, Minas Gerais 31270-901, Brazil; scozeus1@gmail.com; 5 Department of Cell Biology and the Sylvester Comprehensive Cancer Center, University of Miami Miller School of Medicine, Miami, FL 33136, USA; gbarber@med.miami.edu; 6 Laboratory of Immunobiology, Universidade Federal de Santa Catarina, Santa Catarina 88040-900, Brazil; mansurds@gmail.com; 7 Faculdade de Medicina de Ribeirão Preto, Universidade de São Paulo, São Paulo 14049-900, Brazil; thicunha@usp.br; 8 Ann Romney Center for Neurologic Diseases, Brigham and Women’s Hospital, Harvard Medical School, Boston, MA 02115, USA; rmachadorezende@bwh.harvard.edu; 9 Departamento de Fisiologia, Instituto de Ciências Biológicas, Universidade Federal de Minas Gerais, Belo Horizonte, Minas Gerais 31270-901, Brazil; agoufmg@gmail.com

**Keywords:** immune system, DNA sensing, in vivo imaging, immunity, hepatology

## Abstract

Hepatocytes may rupture after a drug overdose, and their intracellular contents act as damage-associated molecular patterns (DAMPs) that lead to additional leukocyte infiltration, amplifying the original injury. Necrosis-derived DNA can be recognized as a DAMP, activating liver non-parenchymal cells (NPCs). We hypothesized that NPCs react to DNA by releasing interferon (IFN)-1, which amplifies acetaminophen (APAP)-triggered liver necrosis. We orally overdosed different knockout mouse strains to investigate the pathways involved in DNA-mediated amplification of APAP-induced necrosis. Mice were imaged under intravital confocal microscopy to estimate injury progression, and hepatocytes and liver NPCs were differentially isolated for gene expression assays. Flow cytometry (FACS) using a fluorescent reporter mouse estimated the interferon-beta production by liver leukocytes under different injury conditions. We also treated mice with DNase to investigate the role of necrosis DNA signaling in IFN-1 production. Hepatocytes released a large amount of DNA after APAP overdose, which was not primarily sensed by these cells. However, liver NPCs promptly sensed such environmental disturbances and activated several DNA sensing pathways. Liver NPCs synthesized and released IFN-1, which was associated with concomitant hepatocyte necrosis. Ablation of IFN-1 recognition in interferon α/β receptor (IFNAR^−/−^) mice delayed APAP-mediated liver necrosis and dampened IFN-1 sensing pathways. We demonstrated a novel loop involving DNA recognition by hepatic NPCs and additional IFN-1 mediated hepatocyte death.

## 1. Introduction

Acetaminophen (APAP) poisoning is the leading cause of acute liver injury in the United States and Europe, accounting for approximately 50% of hepatic liver failure cases [1]. Because pharmacological therapeutic options are restricted to *N*-acetyl-cysteine treatment in the first 12 h after intoxication, liver transplantation in severe cases is the only effective lifesaving procedure. Overall, the therapeutic and supervised use of APAP is safe; however, due to over-the-counter purchase and drug abuse, APAP may become lethally toxic to the liver. Of note, the patient may also suffer from idiosyncratic and intrinsic drug-induced liver injury [2,3,4]. The hepatic injury induced by APAP can be divided into two phases: upon intake and absorption, excessive amounts of the drug can be rapidly bioactivated by hepatocytes (including the CYP2E1 pathway) to toxic intermediates (including *N*-acetyl-*p*-benzoquinone imine, or NAPQI), causing cell swelling (oncosis) and subsequent necrosis [4]. Later, *bona fide* intracellular components abruptly increase their concentration within the extracellular milieu, which at this time point are considered as damage-associated molecular patterns (DAMPs). DAMPs are a complex class of pro-inflammatory molecules that comprise several components, such as Adenosine Triphosphate (ATP), Heat Shock Protein (HSP), High Mobility Group Box 1 (HMGB1), actin and mitochondrial products, as well as genomic and mitochondrial DNA [5]. It is important to consider that different cell lines will harbor different concentrations of intracellular components even under steady state. Thus, upon rupture during necrosis, different intensities of inflammatory processes may be triggered within organs. 

During postnatal growth, the liver undergoes dramatic changes that are characterized by a gradual enhancement of both nuclei number and DNA content per cell [6]. This may be explained by the several cycles of cell division that hepatocytes have, which favor the generation of polyploid cells [7]. Interestingly, such successive division may give rise to tetraploid and octoploid cells harboring one or two nuclei. The rate of polyploid cells increases over the aging process but can also be triggered by cellular stress (hepatectomy), toxic stimulation, and metabolic overload during changes in diet [6,8]. In fact, these tenets hold true for both mouse and humans. In this context, the liver has one of the major populations of polyploid cells within the body, and mature hepatocytes may have up to 16 copies of the genome; a substantial part of cell weight may be due to DNA mass [9]. It is, therefore, reasonable to hypothesize that massive hepatocyte necrosis, such as that observed during APAP overdose, will offer large amounts of extracellular DNA. This induces the immune system activation via a plethora of different DNA sensors that are expressed by these cells, including stimulator of IFN genes (STING), cyclic guanosine monophosphate-adenosine monophosphate synthase (cGAS), Toll-like receptor 9 (TLR9), and others. Recognition of self or exogenous DNA within the membrane via cytoplasmic sensors usually suggests pathogenic invasion [10,11]. However, self-DNA trafficking may occur in both physiological and pathological situations. Upon DNA sensing, several innate immune DNA-sensing pathways trigger an antimicrobial type 1 interferon (IFN) response, which also may include other cytokines (Tumor Necrosis Factor (TNF)-α, Interleukin (IL)-6, IL-1B, MCP-1, and others) [12]. This response, which initially can enhance host protection, could also become damaging if it is improperly activated by self-DNA as occurs during necrosis [13,14].

Here we show that there is “labor division” within the different cell populations in the liver during the acute response to APAP-mediated necrosis. Although hepatocytes bioactivate large amounts of APAP and evolve to necrosis and overt extravasation of DNA to sinusoidal microcirculation, we could not detect DNA sensing by hepatocytes. However, liver non-parenchymal cells—namely neutrophils, macrophages, dendritic cells, and lymphocytes—promptly sensed such environmental disturbances, activating several DNA sensing pathways. In fact, liver non-parenchymal cells synthesized and released significant amounts of type 1 interferon (IFN), which was associated with concomitant maintenance of hepatocyte necrosis. Mechanistically, ablation of type 1 IFN recognition in INFAR^−/−^ mice dampened APAP-mediated liver necrosis and type 1 IFN-related pathways. This elucidated a novel loop involving DNA released by necrotic hepatocytes, recognition by liver sentinel immune cells and additional type 1 IFN-mediated hepatocyte death. This may guide further pharmacological interventions aimed at controlling liver injury by dampening type 1 IFN sensing during acute hepatic necrosis.

## 2. Materials and Methods

### 2.1. Animals

C57BL/6J and IFNAR^−/−^ mice aged 8 to 13 weeks old were purchased from Centro de Bioterismo in Universidade Federal de Minas Gerais (CEBIO-UFMG, Brazil). *Ifnb1*-eYFP mice (B6.129-*Ifnb1*^tm1Lky^/J) were purchased from Jackson (Stock No: 010818). STING^−/−^ mice were obtained from Dr. Glen Barber’s lab [15] and cGAS^−/−^ mice [16] were obtained from Dr. Sergio Costa’s lab. All mice used in this work were sex matched (females) in order to reduce variance and assure significant grades of hepatic injury. Females were chosen based on preliminary experiments that—in our hands—granted more reproducibility and mimicked severe injury cases. Mice were housed under controlled conditions of temperature (24 °C) with a light/dark cycle of 12/12 h, and with chow and water *ad libitum*. The Animal Care and Use Committee at UFMG approved all animal studies (CEUA 377/2016).

### 2.2. Drug-Induced Liver Injury Model

Mice were fasted for 12 h before vehicle or APAP administration (600 mg/kg). Acetaminophen (Sigma-Aldrich, St. Louis, MO, USA) was dissolved in warm saline prior to gavage. Serum alanine aminotransferase (ALT) activity was performed using a kinetic test (Bioclin, Belo Horizonte, Minas Gerais, Brazil). Liver fragments were collected for histology (hematoxylin and eosin staining). IL-1b was quantified in whole liver tissue using enzyme-linked immunosorbent assay (ELISA) kits (R&D Systems Inc., McKinley, Minneapolis, MN, USA).

### 2.3. In Vivo Mouse Imaging

Confocal intravital imaging was performed as previously described [3]. Briefly, mice were anesthetized (i.p.) with a mixture of ketamine and xylazine (Syntec, 60 mg/kg and 15 mg/kg, respectively) and a midline laparoscopy was performed to expose the liver for imaging. Prior to surgery, mice were injected i.v. with 1 µL of Sytox Green, (Invitrogen, Carlsbad, CA, USA) and Phycoerythrin (PE)-conjugated anti-Ly6G (4 µg, eBiosciences, San Diego, CA, USA clone 1A8). Positive labeling was confirmed by injection of matched isotype control (PE-rat anti-mouse IgG). Labeled antibodies and fluorescent probes were diluted in a total volume of 100 µL before injection. Mice were imaged using a Nikon Eclipse Ti (Nikon, Shinagawa, Tokyo, Japan) with an A1R confocal microscope loaded with a spectral detector and XYZ motorized stage. To confirm type 1 IFN production in our Yellow Fluorescent Protein (YFP)-expressing strain, mice were treated with R848—resiquimod (TLR7 and TLR8 agonist); 50 nmol/mouse, i.p., 24 h before imaging.

### 2.4. Primary Murine Hepatocytes

Primary hepatocyte purification was performed as described previously [17,18]. Briefly, mice were treated with APAP and 6, 12, and 24 h after treatment mice were anesthetized and submitted to liver perfusion with collagenase (Sigma-Aldrich, C2139) through the portal vein. After the perfusion, the liver was transferred to a sterile glass container and dissociated with forceps whilst in Williams’ E medium. The cell suspension was filtered through a 40 µm sterile nylon mesh, transferred to a 50 mL tube, and then centrifuged twice at 60× *g* for 3 min at 4 °C. Following centrifugation, cell viability was determined by trypan blue dye. 

### 2.5. Liver Non-Parenchymal Cell (NPC) Isolation

Mice were treated with APAP and 6, 12, and 24 h after treatment the liver was removed, minced into small pieces, and digested using a solution of RPMI medium supplemented with 10% fetal bovine serum and collagenase VIII (Sigma—C2139, 1 mg/mL). After incubation under agitation for 1 h at 37 °C in the incubator, the liver homogenate was filtered through a 70 µm cell strainer to remove undigested tissue. The filtrate was transferred to a 50 mL tube and differentially centrifuged (1st: 300× *g* for 5 min at 4 °C; 2nd and 3rd: 60× *g* for 3 min at 4 °C; and 4th: 300× *g* for 5 min at 4 °C). The pellet was reconstituted for further analysis. For flow cytometry, 5 × 10^5^ cells were used and each sample was read in an Accuri^TM^ C6 cytometer (Becton-Dickson, Franklin Lakes, NJ, USA). FlowJo (FlowJo, LLC, Ashland, OR, USA) and Accuri^TM^ C6 software were used to analyze the results.

### 2.6. RNA Extraction and Real-Time PCR

Total RNA isolation from NPCs and hepatocytes was performed by the Aurum^TM^ Total RNA Fatty and Fibrous Tissue kit (Bio-Rad, Hercules, California, USA) following the manufacturer’s instructions. Total RNA was quantified using NanoDrop^TM^ One (Thermo Fisher Scientific, Waltham, MA, USA) and 1 µg of RNA was used to perform reverse transcription with iScript^TM^ cDNA Synthesis Kit. The resulting cDNA was amplified by PCR with iTaq^TM^ Universal SYBR^®^ Green Supermix (Bio-Rad, Hercules, CA, USA) in a Rotor-Gene Q (Qiagen, Hilden, Germany) (Supplementary Table S1). All primers were selected from PrimerBank (http://pga.mgh.harvard.edu/primerbank/). GAPDH (forward 5′-AGGTCGGTGTGAACGGATTTG-3′, reverse 5′-TGTAGACCATGTAGTTGAGGTCA-3′) was chosen out of six reference genes tested using NormFinder Software [19]. Heatmap was created in MeV software [20,21].

### 2.7. GSH Quantification Assay

C57BL/6, STING^−/−^, and IFNAR^−/−^ mice received APAP (600 mg/kg) by gavage. Two hours after the challenge, the liver was harvested. Then, 150 µL of phosphate buffer saline (pH 6.5) was added to a 150 mg sample. The samples were disrupted with a homogenizer and 300 µL of trichloroacetic acid (12.5%, pH 2.0) was added, mixed, and centrifuged at 6000× *g* for 15 min at 4 °C. For determining glutathione (GSH) levels, 40 µL of supernatant was added to a 96-well microplate, followed by 240 µL of Tris-HCl (Trizma base 0.9M, pH 8). Then, 20 µL of 5,5′-dithiobis(2-nitrobenzoic acid) (0.25 M in methanol + Tris-HCl 1:3) was added to the wells. Color intensity was immediately measured at 415 nm.

### 2.8. Statistical Analysis

Experimental data analysis was performed with one-way analysis of variance (ANOVA with Tukey’s post-hoc test) and Student’s *t*-test provided by Prism 6.0 software (GraphPad Softwares, Inc., La Jolla, CA, USA). All data are given as the mean ± SEM. In vivo experimental groups had at least three mice per group. Data shown are representative of at least two independent experiments. Differences were considered significant when *p <* 0.05.

## 3. Results

### 3.1. APAP Overdose Causes Massive DNA Accumulation within Necrotic Areas and Liver Sinusoids

**Figure 1:**
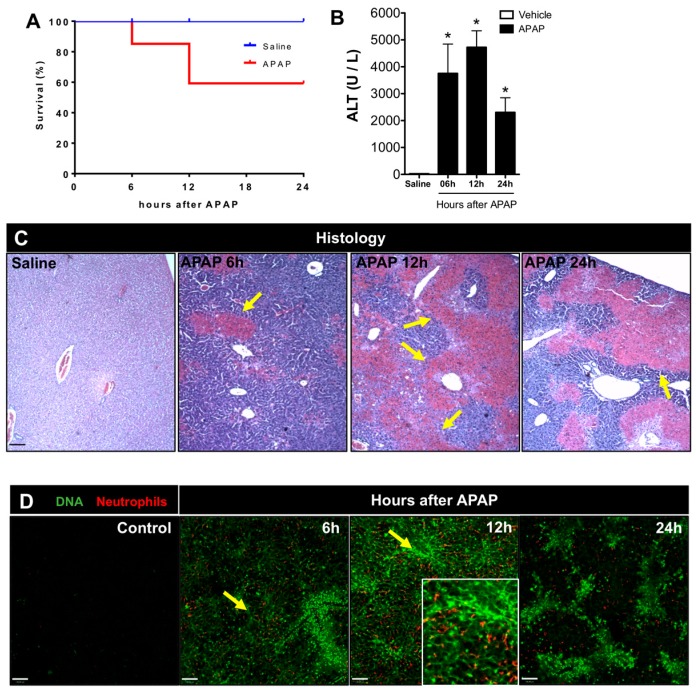
DNA deposition in liver microvasculature during acetaminophen (APAP)-induced injury. (**A**) Survival curve from mice (*n* = 10/group) that were challenged with APAP or saline (control). (**B**) Alanine aminotransferase (ALT) levels in serum to assess hepatic injury at 6, 12, and 24 h after APAP administration. (**C**) Liver histology at different times after APAP administration evidencing hepatic injury; coloration Hematoxylin & Eosin (HE); 40× magnification. Arrows show areas of extensive necrosis; scale bar = 100 μm (**D**) Liver intravital confocal microscopy at different times after hepatic injury; green: Sytox Green; red: anti-Ly6G; scale bar = 300 μm; 100× magnification. (Mean ± SEM; *n* = 4); * *p* ≤ 0.05. Arrows show areas of massive DNA accumulation (necrosis), and the insert in the 12-h panel shows accumulation of neutrophils within DNA-rich areas (magnification 4×).

**Figure 2:**
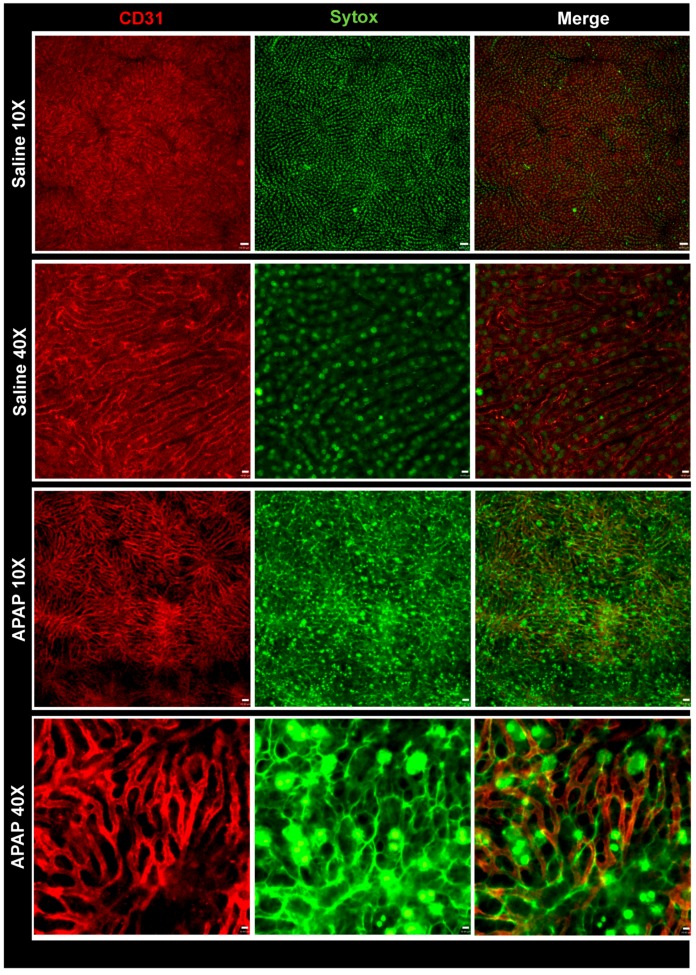
Intravital confirmation of DNA deposition in liver microvasculature during APAP-induced injury. Mice were treated with APAP and after 12 h were prepared for intravital microscopy. Note the extensive extravascular DNA deposition within the vessels. Liver intravital microscopy from a saline-treated mouse (10× magnification) showing (**A**) sinusoids, (**B**) DNA staining and (**C**) merged channels. Liver intravital microscopy from a saline-treated mouse (40× magnification) showing (**D**) sinusoids, (**E**) DNA staining and (**F**) merged channels. Liver intravital microscopy from an APAP-challenged mouse 10× magnification showing (**G**) sinusoids, (**H**) DNA deposition and (**I**) merged image. Liver intravital microscopy from an APAP-challenged mouse 10× magnification showing (**J**) sinusoids, (**K**) DNA deposition and (**L**) merged channels. Arrows show intravascular areas of DNA accumulation; scale bar = 50 μm (10×) and 13 μm (40×). Green: Sytox Green; red: Phycoerythrin-conjugated anti-CD31.

To investigate the dynamics of DNA release and its effects on different hepatic cells, we first established a mouse model of drug-induced liver injury by administering an APAP overdose. As shown in Figure 1A, 20% of the APAP-challenged mice died 6 h after gavage, and significantly higher levels of alanine aminotransferase (ALT) were detected in the serum (Figure 1B). Since the APAP dose was administered *in bolus*, higher drug levels reached the systemic circulation. Concomitantly, in histopathology, areas of centrilobular necrosis were observed (Figure 1C, yellow arrows) together with significant DNA deposition within the liver microvasculature in vivo (Figure 1D). Of note, the majority of extracellular DNA was found lining the sinusoidal lumen (arrows), and several centrilobular hepatocytes were stained by Sytox Green (live cell impermeant DNA-staining probe; Figure 1D), indicating that the hepatocyte cell membrane had already started to lose integrity. Using three-dimensional reconstruction, we confirmed that extracellular DNA released during necrosis had accumulated in islets within hepatocytes as large areas of DNA deposition. Also, when we counterstained liver sinusoids in vivo with anti-CD31, we could clearly observe that DNA had also accumulated within the vessel lumen (Figure 2 and Supplemental Movie 1). In addition, intravital microscopy revealed that neutrophils accumulated within sinusoids (insert; Figure 1D), evidencing a classic feature of acute liver inflammatory response after the chemical challenge. At 12 h after APAP overdose, we observed 40% lethality and higher serum ALT levels; centrilobular necrosis was also detected (Figure 1A–C). In fact, extracellular DNA deposition had largely increased, together with elevated numbers of infiltrating hepatic neutrophils. At 24 h after APAP challenge, despite that the ALT serum levels had decreased, we still observed, histologically, several neutrophils and large necrotic areas, which were confirmed by extracellular DNA staining in intravital microscopy (Figure 1D). Taken together, we established that APAP-induced liver injury caused a marked DNA release and accumulation, allowing further investigations on the role of DNA recognition within the liver cellular microenvironment.

### 3.2. Liver Non-Parenchymal Cells Express Higher Levels of DNA Sensors than Hepatocytes during Homeostasis

**Figure 3:**
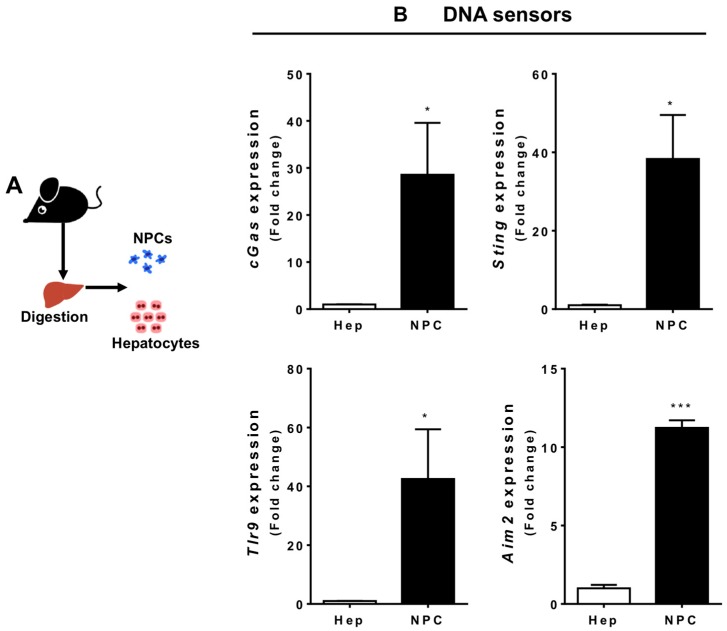
DNA sensor expression in different liver cell populations. (**A**) Scheme showing two groups of isolated hepatic cells—parenchymal (hepatocytes) and non-parenchymal (NPCs) cells—from healthy mice. (**B**) Gene expression comparison of different DNA and cytokine sensors between these two populations. The graphs represent the mean expression of selected genes relative to hepatocytes (ΔΔCT). (Mean ± SEM; *n* = 4); * *p* ≤ 0.05 and *** *p* ≤ 0.001 compared to hepatocytes.

The massive DNA accumulation triggered by APAP overdose led us to investigate which populations of hepatic cells sense and respond to extracellular DNA. For this, we isolated hepatocytes and liver non-parenchymal cells (NPCs) to determine the expression of the different DNA sensors, including cGAS, STING, TLR9, and AIM2 (Figure 3A). Of note, we established a protocol that allowed isolation of both hepatocytes and liver immune cells from mice that were challenged in vivo with APAP—instead of the classic in vitro incubation protocols—to guarantee better translation to the actual liver microenvironment. In this case, only viable liver cells were used throughout the analysis. We found that liver NPCs expressed higher levels of all DNA sensors investigated in comparison to hepatocytes, whereas hepatocytes expressed lower levels of all these sensors (Figure 3B). This suggests that liver NPCs are the main DNA sensors within the liver microenvironment.

**Figure 4:**
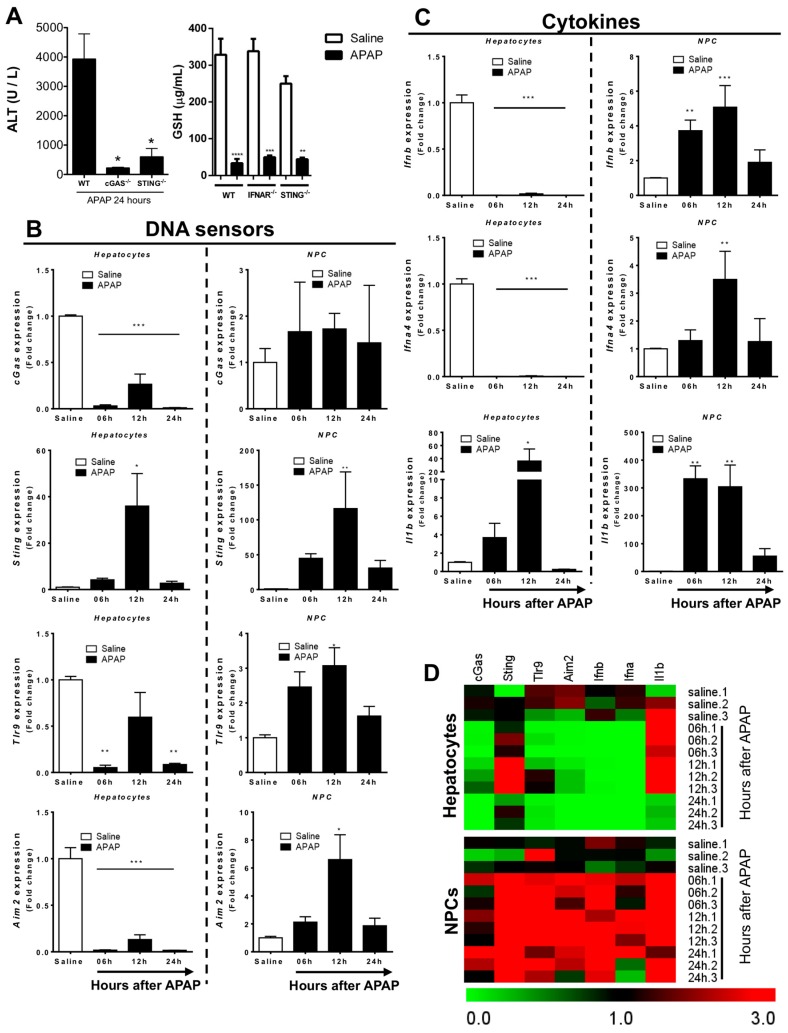
Evaluation of DNA sensor expression in different hepatic populations during APAP injury. (**A**) Absence of DNA sensing pathways cGAS, IFNAR and stimulator of IFN gene (STING) completely protected mice against APAP-induced liver injury, but with no detectable changes in GSH levels during APAP challenge. (**B**) Gene expression of different DNA sensors and (**C**) cytokines in NPCs and hepatocytes cells after hepatic injury induction; the relative expression was done using the control (saline) population of each group as a reference. (**D**) Heatmap showing variations in gene expression at different times after induction of hepatic injury; green = decreased expression; black = no variation; red = increased expression. (Mean ± SEM; *n* = 4); * *p* ≤ 0.05, ** *p* ≤ 0.01, *** *p* ≤ 0.001 and **** *p* ≤ 0.0001. The graphs represent the mean expression of selected genes relative to saline hepatocytes (ΔΔCT).

Once we had established the homeostatic expression of DNA sensing pathways in liver NPCs, we next investigated how different liver cell populations react to extracellular DNA released in response to APAP overdose. For this, we challenged mice with APAP and after different time points, hepatocytes and liver NPCs were isolated for further gene expression analysis. Mice deficient in STING or cGAS were completely resistant to APAP-induced liver injury, providing a strong link between DNA-cGAS-STING pathways in the pathogenesis of acute liver injury (Figure 4A). Of note, these mice had similar hepatic glutathione levels. In addition, both knockout strains were fully able to bioactivate APAP since we observed a major depletion in hepatic GSH levels 2 h after challenge [22] (Figure 4A). In addition, we found a significant increase in *Sting* expression by hepatocytes 12 h after the APAP challenge (Figure 4B), which coincided with the peak of DNA release imaged by intravital microscopy. However, TLR9, AIM2, and cGAS were downregulated in these cells at all the time points evaluated (Figure 4C), suggesting that despite elevated *Sting* expression, DNA sensing by hepatocytes may be dampened during APAP challenge due to a reduction of the key intracellular adaptors or other signaling pathways. In contrast, liver NPCs consistently upregulated DNA sensing pathways following APAP treatment, reaching higher expression levels 12 h after overdose (Figure 4C). Additionally, we observed that pathways related to type 1 IFN production (*Ifnb* and *Ifna4*) were upregulated specifically in liver NPCs but not in hepatocytes. In fact, APAP induced downregulation in the expression of type 1 IFN pathways in hepatocytes and upregulation in IL-1βproduction in hepatocytes and NPCs (Figure 4C). Thus, APAP overdose led to an enhancement in the expression of DNA sensing and inflammatory pathways in different hepatic populations with a significantly more pronounced effect in liver NPCs (Figure 4D).

### 3.3. Liver Non-Parenchymal Cells Release Type 1 IFN during APAP Overdose, Which Is Concomitantly Sensed by Hepatocytes

**Figure 5:**
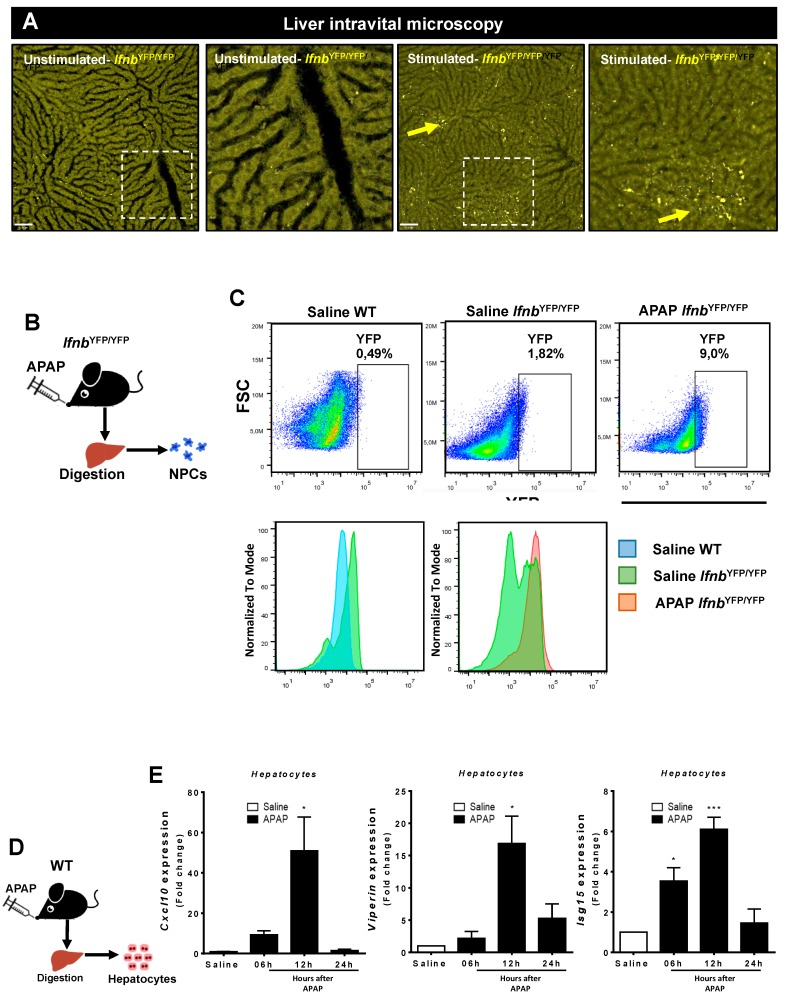
Interferon (IFN)-I production in liver during APAP injury. (**A**) Confocal intravital microscopy of IFN-beta reporter mouse (IFN^YFP/YFP^) showing enhancement of IFN-beta expression upon stimulation in vivo. Scale bar = 60 μm and inserts are derived from a 4× magnification. Yellow arrows show spots of IFN-beta accumulation. (**B**) Scheme showing parenchymal cell isolation (hepatocytes) in mice treated with APAP (600 mg/kg). (**C**) Flow cytometry for the evaluation of IFN-I production by non-parenchymal liver cells in IFN^YFP/YFP^ mice. (**D**) Scheme showing the non-parenchymal cell isolation from IFN^YFP/YFP^ mice 12 h after treatment with APAP (600 mg/kg). (**E**) Evaluation of IFN-I-regulated gene expression in hepatocytes after hepatic injury induction; the relative expression was done using the control (saline) hepatocytes as a reference. (Mean ± SEM; *n* = 4); * *p* ≤ 0.05 and *** *p* ≤ 0.001 compared to control hepatocytes.

Once we had established that liver NPCs are the primary DNA sensors within the liver, we sought to directly visualize the dynamics of type 1 IFN production during APAP-induced liver injury. To specifically address this question, we used a mouse strain in which the production of type 1 IFN is under the control of yellow fluorescent protein (IFN-beta^YFP/YFP^ mice; Figure 5A,B). To confirm that this mouse strain was able to report IFN-b under stimulation, we challenged the mice in vivo with a systemic injection of R848 (Resiquimod, a TLR7 and TLR8 agonist). As shown in Figure 5A, in comparison to non-stimulated mice, R848 injected mice had several YFP-expressing cells within the liver (arrows), validating our IFN-reporter model. Under our imaging setup, even lethal doses of APAP did not allow the visualization in vivo of type 1 IFN production, despite that it was clearly seen under flow cytometry. We found that ~2% of NPCs from IFN-beta^YFP/YFP^ mice constitutively expressed type 1 IFN under baseline conditions and that the frequency of IFN-beta was almost 5 times greater after APAP overdose (~9%; Figure 5B,C). In fact, while controls had a higher population of IFN-beta^low^ cells, APAP-treated IFN-beta^YFP/YFP^ mice were predominantly IFN-beta^high^ NPCs (Figure 5C). This suggests that liver NPCs not only express higher levels of DNA sensing pathways but also produce significant levels of IFN-beta during APAP overdose.

We next hypothesized that type 1 IFN released by NPCs are sensed by hepatocytes during acute liver injury. In line with this, we found that isolated hepatocytes significantly upregulated different genes involved in type 1 IFN cell activation, including *Cxcl10*, viperin (*Rsad2*), and *Isg15* after APAP overdose in vivo (Figure 5D,E). Interestingly, all of these genes were already expressed 6 h after overdose but were markedly increased 12 h after APAP challenge, concomitant with the peak of hepatocyte necrosis, extracellular DNA release, and activation of liver NPCs. Of note, expression values returned to baseline 24 h after APAP administration. Thus, DNA sensing by liver NPCs triggers type 1 IFN production, which is subsequently sensed by hepatocytes during massive liver necrosis.

### 3.4. Absence of Extracellular DNA Abrogates IFN-Beta Production and Sensing within the Liver during APAP-Induced Injury

**Figure 6:**
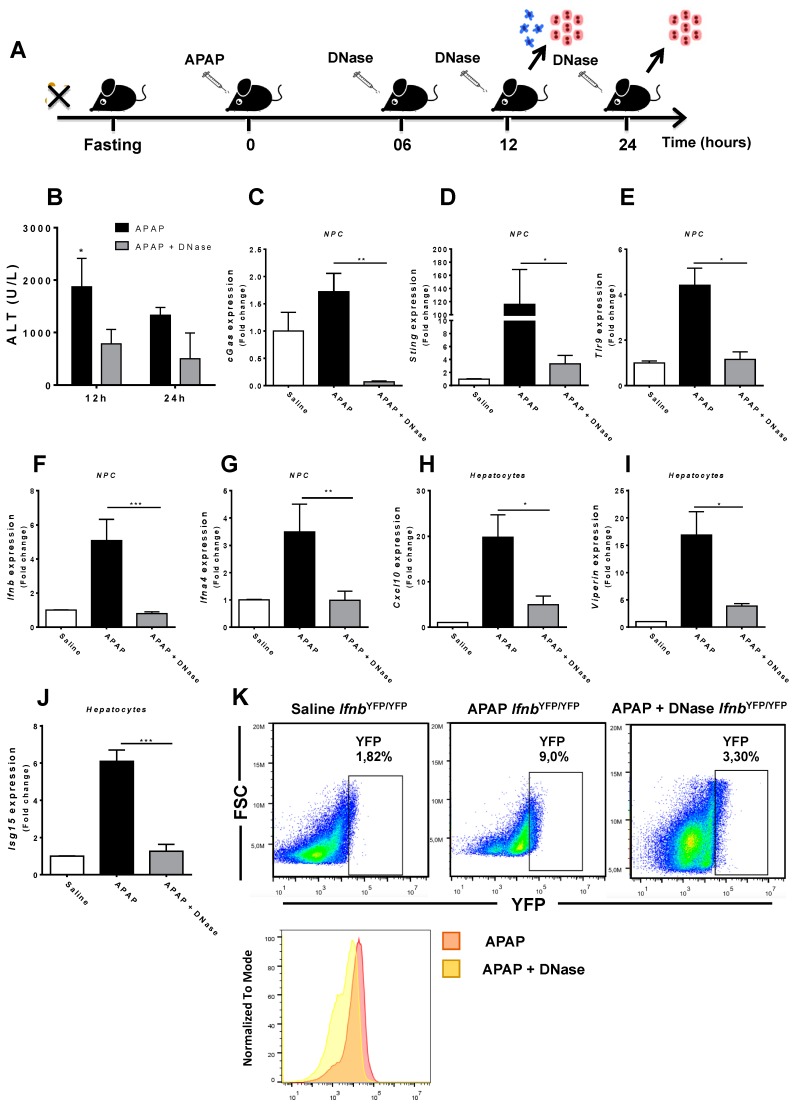
DNase treatment after hepatic injury induction. (**A**) Scheme showing APAP treatment time points (600 mg/kg) and DNase (1000 U/L) in mice over time. (**B**) ALT serum levels to assess hepatic injury at 12 and 24 h after DNase treatment. (**C**–**G**) Gene expression of different sensors in hepatic non-parenchymal cells between treated and non-treated groups with DNase. Cells were collected 12 h after administration of APAP (600 mg/kg) and relative expression was done using control NPCs (saline) as a reference. (**H**–**K**) Gene expression of different sensors in hepatocytes between DNase-treated and non-treated groups. (**K**) Flow cytometry to evaluate IFN-I production by non-parenchymal liver cells in DNase-treated and untreated groups. Cells were collected from IFN^YFP/YFP^ mice 12 h after administration of APAP (600 mg/kg). (Mean ± SEM; *n* = 4); * *p* ≤ 0.05, ** *p* ≤ 0.01 and *** *p* ≤ 0.001. The relative gene expression was done using saline hepatocytes as a reference.

To confirm that extracellular DNA released during necrosis was fuelling liver NPC activation and hepatocyte necrosis, we treated mice during the evolution of APAP-induced injury with a commercially available DNase (Sigma-Aldrich) (Figure 6A). Interestingly, similar formulations have already been FDA approved for other medical conditions, for instance, in the treatment of pulmonary fibrosis (ex. Pulmozyme^®^, Roche, Basel, Switzerland). Systemic DNase treatment significantly rescued liver injury in APAP-treated mice even when administered 6 h after intoxication (Figure 6B). Consistent with this, DNA removal due to DNase administration completely abolished the expression of several DNA sensing pathways in liver NPCs in mice challenged with APAP (Figure 6C–G). Moreover, we found a significant reduction in gene levels involved in the production of type 1 IFN (*Ifnb* and *Ifna4*), which was confirmed by a massive dampening in IFN-beta synthesis by liver NPCs in vivo (Figure 6K). Strikingly, the absence of DNA signaling and type 1 IFN secretion after DNase treatment was accompanied by a significant reduction in type 1 IFN signaling pathways in hepatocytes (Figure 6H–J), providing a strong link between necrosis, DNA release/sensing, and type 1 IFN signaling during drug-induced liver injury.

### 3.5. Lack of Type 1 IFN Signaling Protects Mice from APAP-Induced Liver Injury

**Figure 7:**
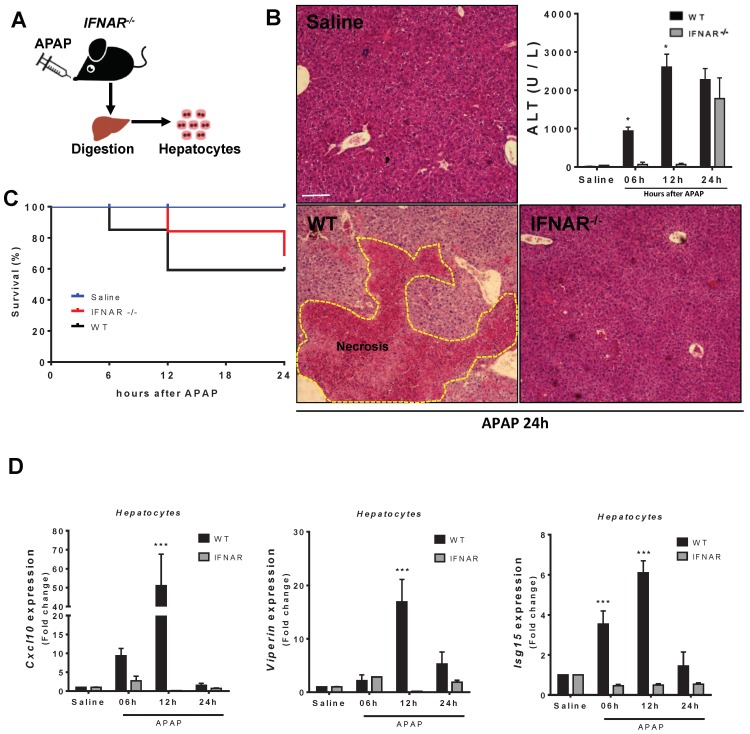
IFN-I production aggravates hepatic injury. (**A**) Scheme showing hepatocyte isolation from IFNAR^−/−^ mice challenged with APAP. (**B**) Liver histology and serum ALT levels of IFNAR^−/−^ mice 24 h after APAP (600 mg/kg) administration (HE staining). (**C**) Survival rate in IFNAR^−/−^ mice after APAP (600 mg/kg) administration. (**D**) Expression of viperin, *Isg15* and *Cxcl10* in IFNAR^−/−^ mouse hepatocytes in comparison to wild-type cells 6, 12, and 24 h after APAP administration. (Mean ± SEM; *n* = 5); * *p* ≤ 0.05 and *** *p* ≤ 0.001. Scale bar = 200 μm.

**Figure 8:**
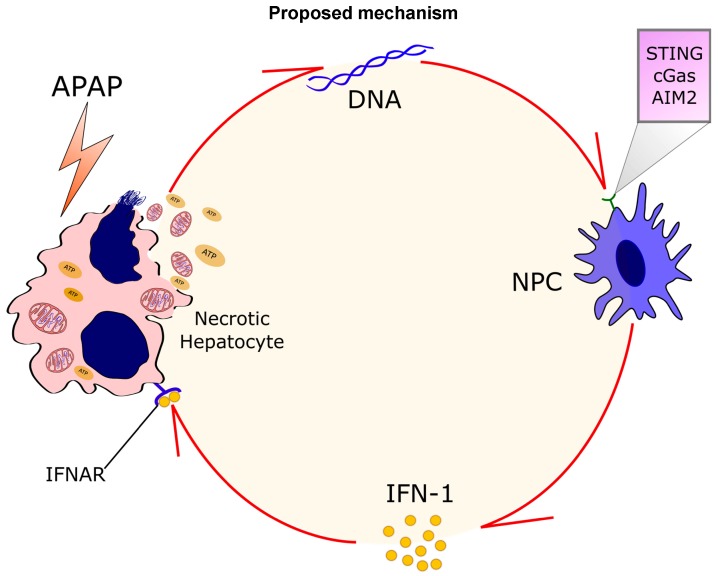
Proposed mechanism—APAP-mediated liver necrosis leads to massive DNA release and deposition within the liver. IFN-I-mediated oxidative stress caused by liver NPCs is a master mediator of type 1 IFN-induced liver damage. Therefore, controlling innate immune response during APAP-mediated injury may dampen APAP-triggered liver necrosis, paving a new road to further clinical interventions.

To expand our investigation on type 1 IFN signaling during APAP-mediated liver injury, we challenged mice that had both interferon alpha- and beta-receptors deleted (IFNAR^−/−^; Figure 6A). IFNAR^−/−^ mice had a delayed response to injury (Figure 7B,C) in comparison to wild-type mice. However, although the mortality rate in IFNAR^−/−^ mice in the initial time points was lower (~20%), they evolved to a similar lethality as the wild-type mice even with liver histologic analyses showing no signs of necrosis 24 h after injury (Figure 7B,C). Moreover, significantly lower serum ALT levels in IFNAR^−/−^ mice were found between 6 and 12 h after injury but increased to levels comparable to wild-type mice 24 h after APAP treatment (Figure 7B). Importantly, type 1 IFN-induced genes, such as viperin, *Isg15*, and *Cxcl10*, were dramatically reduced in IFNAR^−/−^ mice, particularly 12 h after injury (Figure 7D). Thus, type 1 IFN signaling appears to be critical for the initial phase of APAP-induced liver injury, but alternative amplification mechanisms took place at later time points of APAP toxicity. A proposed mechanism for the amplification of DNA-mediated IFN-1 release is depicted in Figure 8.

## 4. Discussion

We revealed a novel pathway that congregates direct toxic injury, DNA release from necrotic hepatocytes, and activation of a cascade that involves STING and cGAS-mediated self-DNA sensing from liver non-parenchymal cells. These pathways culminate in further type 1 IFN production and amplification of liver injury. These data have a substantial impact on our understanding of why patients subjected to drug-induced liver injury evolve to worse hepatic necrosis even when the initial hit is removed. In this direction, our data shed light on alternative therapeutics directed to not only dampen the direct effect of toxic mechanisms of medicines but also in the amplification loop that may be triggered due to activation of the liver immune system.

Here we expanded our previous findings that described a massive deposition of extracellular DNA within the injured liver, and despite neutrophil depletion during disease, extracellular DNA was still widely present [23]. This supports the hypothesis that hepatocytes could be a major source of liver-accumulated DNA. In this context, since neutrophil depletion did not affect DNA accumulation within the liver [23], the participation of neutrophil extracellular traps (NETs) to sterile liver injury seems to be minor or absent. It is worth mentioning that part of the extracellular DNA released from necrosis would be washed out of the vessels due to the blood flow, and also another part may be degraded by serum DNase I. Therefore, the actual amount of DNA released during necrosis may be even higher than we are able to detect using our in vivo system. Of note, blood flow within the liver is relatively slow in comparison to other organs, which may account for the significant amount of DNA retention within the hepatic microcirculation. We also previously demonstrated that acute DNA removal by intravenous DNase or TLR9 depletion/pharmacological blockage specific on neutrophils significantly abrogated APAP-mediated liver injury [23]. Thus, the questions that still remain to be answered after these studies are (i) which specific cell subtypes are sensing extravascular DNA and, considering the plethora of intracellular pathways that can recognize and sense DNA, (ii) which of these sensors are crucial to type 1 IFN production and amplification of liver injury.

The mechanisms behind how type 1 IFN could enhance hepatocyte injury are still elusive. Recently, it was reported that downregulation of Sod1 boosts oxidative liver stress in mice [24]. In this context, dampening type 1 IFN signaling would help in rescuing liver damage during viral infections. Therefore, type 1 IFN signaling may be crucial for triggering oxidative stress and enhancing organ injury during situations when DNA is being recognized by the cells in either an infectious or a sterile context. We also hypothesized that due to the disproportional amount of DNA that hepatocytes normally harbor, these cells would not express functional DNA-sensing pathways. This may be supported by reports that have described lower STING expression by hepatocytes under steady state [25]. In a broad sense, avoiding unwanted recognition of the enormous amount of self-DNA would prevent recurrent undesired inflammatory responses. However, reduced expression of DNA-sensing pathways by hepatocytes may impact the liver’s ability to control DNA viruses, including hepatitis-B virus [25,26]. Here, we have demonstrated that upon inflammation—which usually occurs during infections—hepatocytes may upregulate STING expression during the acute phase, and despite STING expression in the steady state not being significant, triggering of inflammatory responses may shift such latency to a pro-responsive state. Whether this behavior is effective in enhancing anti-viral cascades is still elusive.

The massive production of DNA-triggered type 1 IFN during APAP inflammation also raises other concerns. Despite new classes of drugs emerging to treat viral hepatitis, older therapeutic strategies are still common in low-income countries. These strategies are based on the administration of alpha-interferon. Not surprisingly, this leads to an extensive number of adverse side effects, including headache, fatigue, fever, arthralgia, and others. These adverse events usually require dose modification or even discontinuation of therapy in 2% to 10% of patients [27]. Despite this therapeutic line becoming obsolete, understanding how to modulate type 1 IFN side effects in the liver may help prevent putative side effects in patients that still do not have access to modern drugs. The major part of the side effects due to type 1 IFN administration relies on the downregulation of superoxide dismutase 1, which catalyzes the removal of free hepatic superoxide radicals, fuelling hepatocyte necrosis and morbidity in patients. In fact, IFNAR^−/−^ mice had a delayed pattern of injury progression in comparison to wild-type mice. It is well established that liver injury due to APAP overdose arises mainly from two pathways: direct injury due to drug toxicity and collateral injury due to an overt inflammatory response that usually takes place at later time points (12–24 h post challenge). Therefore, we propose that the absence of type 1 IFN signaling might dampen the later phases of injury amplification, probably not interfering with ALT serum levels. In combination with our in vivo and gene expression data, we believe that a lack of inflammation amplification may decrease the magnitude of liver necrosis. Here, we expanded these findings—using another clinical model—to suggest that type 1 IFN might be extremely hepatotoxic since ablation of its signaling pathway protected mice against collateral necrosis.

## 5. Conclusions

Our data propose a novel cascade where sterile cell death leads to massive DNA deposition within the liver, and that type 1 IFN-mediated oxidative stress, governed mainly by liver NPCs, is a master cascade of type 1 IFN-induced liver damage. In addition, we provide strong evidence that controlling innate immune response during APAP-mediated injury may hold fruitful research venues for adjuvant treatment of drug-induced liver injury.

## Supplementary Materials

The following are available online, Supplemental Movie S1: Three-dimensional rendering of liver intravital microscopy showing sinusoids (in red) and extracellular DNA (in green). Table S1: List of primers used in the study.
