# Supplementary material for: Liver Immune Cells Release Type 1 Interferon Due to DNA Sensing and Amplify Liver Injury from Acetaminophen Overdose
Source: Cells. 2018 Jul 27;7(8):88. doi: 10.3390/cells7080088 (PMC6115735; doi:10.3390/cells7080088)
Supplement: Supplementary file 1 [file cells-07-00088-s001.zip › Table S1.pdf]

Table S1. List of primers used in the study.

Gene	Primer	Sequence (5'-3')
Gapdh	Forward	AGGTCGGTGTGAACGGATTTG
	Reverse	TGTAGACCATGTAGTTGAGGTCA
18s	Forward	CGTTCCACCAACTAAGAACG
	Reverse	CTCAACACGGGAAACCTCAC
Rpl4	Forward	CCGTCCCCTCATATCGGTGTA
	Reverse	GCATAGGGCTGTCTGTTGTTTTT
Sdha	Forward	GCGGTGGTCACCTTGATCC
	Reverse	CCTCTGTAGAAGCGTCTGAATG
Hprt	Forward	TCAGTCAACGGGGGACATAAA
	Reverse	GGGGCTGTACTGCTTAACCAG
Actb	Forward	GGCTGTATTCCCCTCCATCG
	Reverse	CCAGTTGGTAACAATGCCATGT
Mb21d1 (cGAS)	Forward	GAGGCGCGGAAAGTCGTAA
	Reverse	TTGTCCGGTTCCTTCCTGGA
Tmem173 (STING)	Forward	GGTCACCGCTCCAAATATGTAG
	Reverse	CAGTAGTCCAAGTTCGTGCGA
Aim2	Forward	GTCACCAGTTCCTCAGTTGTG
	Reverse	CACCTCCATTGTCCCTGTTTTAT
Ifna4	Forward	TGATGAGCTACTACTGGTCAGC
	Reverse	GATCTCTTAGCACAAGGATGGC
Ifnb	Forward	TGAATGGAAAGATCAACCTCACCTA

	Reverse	CTCTTCTGCATCTTCTCCGTCA
Il1b	Forward	GCAACTGTTCCTGAACTCAACT
	Reverse	ATCTTTTGGGGTCCGTCAACT
Cxcl10	Forward	CCAAGTGCTGCCGTCATTTTC
	Reverse	GGCTCGCAGGGATGATTTCAA
Rsad2 (Viperin)	Forward	TGCTGGCTGAGAATAGCATTAGG
	Reverse	GCTGAGTGCTGTTCCCATCT
Isg15	Forward	GGTGTCCGTGACTAACTCCAT
	Reverse	TGGAAAGGGTAAGACCGTCCT
Tlr9	Forward	ATGGTTCTCCGTCGAAGGACT
	Reverse	GAGGCTTCAGCTCACAGGG

Heatmap cq values

Hepatocytes

**Gene**	**Group**	**Quantification cycle (Cq)**	
		**Mean**	**Range**
Mb21d1 (cGAS)	Saline	26.14	25.03-27.11
	APAP 06h	29.74	29.11-30.61
	APAP 12h	30.06	30.81-31.56
	APAP 24h	31.17	29.50-30.73

Tmem173 (STING)	Saline	31.69	26.91-29.24
	APAP 06h	27.87	29.23-35.12
	APAP 12h	29.34	28.51-30.45
	APAP 24h	28.79	28.48-29.23
Tlr9	Saline	31.05	29.70-33.05
	APAP 06h	34.09	32.89-35.72
	APAP 12h	34.17	33.44-35.05
	APAP 24h	33.01	32.78-33.22
Aim2	Saline	23.41	22.60-25.95
	APAP 06h	27.50	27.13-27.81
	APAP 12h	28.52	27.87-29.51
	APAP 24h	27.71	27.27-28.11
Ifnb	Saline	29.12	27.89-29.86
	APAP 06h	28.65	28.21-29.09
	APAP 12h	24.93	23.70-25.63
	APAP 24h	23.61	22.35-25-39
Ifna4	Saline	29.03	28.34-29.53
	APAP 06h	30.22	29.19-31.72
	APAP 12h	25.38	23.19-27.08
	APAP 24h	26.53	23.10-28.48
Il1b	Saline	26.77	25.03-29.21
	APAP 06h	23.63	22.70-24.87
	APAP 12h	23.50	21.78-25.43

	APAP 24h	27.29	27.09-27.45

Heatmap cq values

NPCs

Gene	Group	Quantification cycle (Cq)	
		Mean	Range
Mb21d1 (cGAS)	Saline	24.68	23.35-26.71
	APAP 06h	21.25	24.03-25.54
	APAP 12h	21.64	19.74-24.36
	APAP 24h	19.65	16.54-21.46
Tmem173 (STING)	Saline	29.62	28.84-31.15
	APAP 06h	25.54	24.56-26.34
	APAP 12h	21.55	20.81-22.12
	APAP 24h	22.02	19.56-23.51
Tlr9	Saline	28.87	28.27-29.29
	APAP 06h	28.99	28.59-29.43
	APAP 12h	26.09	23.30-29.29
	APAP 24h	25.36	24.19-25.97
Aim2	Saline	22.94	22.67-23.45
	APAP 06h	23.29	22.72-24.12
	APAP 12h	19.49	16.44-24.65
	APAP 24h	19.36	17.22-20.93
Ifnb	Saline	29.12	27.89-29.86

	APAP 06h	28.65	28.21-29.09
	APAP 12h	24.93	23.70-25.63
	APAP 24h	23.61	22.35-25.39
Ifna4	Saline	29.03	28.34-29.53
	APAP 06h	30.22	29.19-31.72
	APAP 12h	25.38	23.19-27.08
	APAP 24h	26.53	23.10-28.48
Il1b	Saline	29.93	29.20-31.14
	APAP 06h	23.75	22.19-26.22
	APAP 12h	22.47	19.33-24.00
	APAP 24h	21.08	21.35-24.41
